# On the Use of Electrolysis in Stenosis of the Cervix Uteri

**Published:** 1897-04

**Authors:** A. S. Wolff

**Affiliations:** State Quarantine Officer, Brownsville, Texas


For the Texas Medical Journal.

### ON THE USE OF ELECTROLYSIS IN STENOSIS OF THE CERVIX UTERI.

BY A. S. WOLFF, M. D., STATE QUARANTINE OFFICER, BROWNSVILLE, TEXAS.

NOTHING new is here claimed; I only desire to add my testimony to the efficacy of electrolysis and its therapeutic value in stenosis of the cervix uteri.

In stricture of the urethra, æsophagus, lachrymal canal, eustachian tube, etc., etc., etc., it is fairly well conceded. Nevertheless, serious doubt is entertained by some as to the success of those who have recorded successful cases by the electrolytical method, but principally from some whose attention has not been called to the subject, or from others who failed in their attempts, in consequence of their want of the technical knowledge, or badly constructed instruments, and hence were defeated.

For certain pathological conditions of the cervix, no claim is made for the therapeutic value of electrolysis, viz: cicatrices—tumors—cancers, or a stenosis depending on great deviations.

Some twenty-six years ago, while in practice in the State of New York, and during my incumbency as chief medical officer of the New York Clinton State Prison Dannemora, I met my old friend, Robert Newman, M. D., at the annual meeting of the New York State Medical Society, of which we have been members for a great many years.

Dr. Newman, at that time, read a paper on the use of electrolysis in cases of stricture of the urethra.

After the adjournment, I went with him to the city, and remained with him for the purpose of acquainting myself with the technique and treatment. I soon realized its great advantages for the purpose, and under his personal instructions and supervision, performed several operations for stricture of the urethra. Since that time, from 1870 up to date, forty-one cases were successfully operated, with invariable success, and up to now have had no occasion to alter my method.

While writing this paper, Dr. Newman was kind enough to forward the *Philadelphia Times and Register*, of the 12th of January, 1893, edited by Wm. F. Waugh, M. D., containing the records of good and reliable authorities on the subject, who, after painstaking investigations, describe hundreds of cases, and add their positive testimony, backed by documentary evidence, and unequivocally give expression to the value of electrolysis in stricture of the urethra.

I only wish to emphasize their experience by adding my own forty-one cases of stricture in their statistics, and in spite of the warfare against Dr. Newman's cathodal electricity, it has come to stay.

Dr. Newman was the first, as far as I know, who developed this branch of electro-therapeutics. It is true, Crussel, in 1839, Melez, Tripier, Wildebrand in 1867, employed the method, but I have failed to find a single case of stenosis of cervix treated by electrolysis, at least in the literature or text books accessible to me. It is, however, probable that others have used the method, and antedated my own. "There is nothing new under the sun." If so, I willingly yield them precedence to prior claim. However, I repeat I find no record of a single case.

The only cases which come near it, are those recorded by Dr. A. P. Lauthorn Smith, in the *Canada Medical Record*, and reproduced in the *Annals of Hygiene*, January number, 1893.

But in these cases the galvanic current is used for cases of dysmenorrhœa, depending on some intra-uterine pathological condition, after curetting had failed, and not for dysmenorrhœa depending on stenosis of the cervix.

Many cases of dysmenorrhœa and sterility are associated with narrowing of the canal of the cervix. To relieve this, several means have been devised. Among the earliest, discission, as done by the late Dr. Marion Sims, recommended in his valuable work on uterine surgery; also done by Sir James Simpson. That method is almost entirely abandoned, as not only dangerous, but because attended with poor success; in spite of the glass plug, or the hard rubber dilators, to keep the canal patulous, union is apt to take place, and the patient returns with her old complaint as bad as ever.

Dilatation for the atresia, by aid of a sponge or laminaria, have both strong advocates, but apart from the annoyance incident on the method, and despite of the utmost antiseptic precaution, infection cannot always be prevented. Hausman found that after the sponge had been an hour and a half in situ the tents were covered with epithelium, the secretions discharged contained micrococci and compounds of sponge.

Rapid and forcible dilatation by instruments have their adherents. Dr. Goodell, of Philadelphia, by a modification of Elinger's dilator, has popularized that operation. Solid dilators, introduced closed, and then separated, finds still advocates, but must be deprecated as more or less dangerous, even if employed by the most accomplished and dexterous gynecologist.

Acids and caustics have had their day. Of all means employed, this always appeared to me most objectionable. Their effects cannot always be accurately measured, or so limited in their action as to be wholly under the surgeon's control. All the above methods have their grave faults, the same objection of any is applicable to them all, and worse than all, the medical profesfession is yet divided in its opinion as to which method is the least harmless. Dilatation is uselesss in view of the fact that the tissues of the cervix show an obstinate tendency to contract again, even after thorough dilatation has been done. The immediate result may be good, but ultimately the canal contracts again; very little or any good has been accomplished. Unless the dilatation to be done is slight, it must be done under anæsthetics, which, to a certain extent, must be a drawback.

Electrolysis is a method not only devoid of danger, but has not any of the other disadvantages, and is vastly superior to all.

Stricture of the cervical canal can be as easily relieved as can stricture of the urethra. In addition of curing the atresia, the galvanic current has the advantage of acting beneficially upon the catarrhal discharges, always a source of trouble in these cases, caused by structural alterations in consequence of venous obstruction. The chemical action of the current upon the tissues is of the utmost benefit, and soon stops entirely the discharge.

For practical purpose, one case, if it can be clearly told and to the point, will convey as much information as will the statistical record of fifty, presenting about the same pathological conditions; hence, to save space and reiteration, I only report the following case, as evidence of the great and indisputable value of electrolysis in the most severe cases of stenosis of the cervix:

Miss Ann M. came under my observation on the 14th of November, 1890; 22 years of age, single, of American parents, free from diathesis, pale, habitually constipated, complains of much headache, sleeps fairly well, appetite bad.

Her sister, who brought her to the office, is an intelligent married woman, and who gives me the following information:

Ann's catamenia appeared when about 13 years of age by a slight show. Up to her 17th year her periods were very irregular, at times scant, or only a show; often stopped for four or five months together; always suffering excruciating pain on the appearance of some scant menses; a few days before the menstrual nisus, becomes hysterical, and has fits; after one or two days more of agony, a scanty discharge appears, but instead of being red, is brown, and then black; after a day or two it becomes brown, and then lighter, never red; after much suffering, when some of retained menses are with difficulty expelled, partial relief follows.

Unfortunately for the poor girl, I neglected to make a vaginal examination at that time. Had I done so, I think much of her subsequent suffering might have been avoided. I made a wrong diagnosis, assuming that it was a simple case of irregular menstruation, physiological in its character.

Prescribed hip baths and injections of—

Liq. ammon..............................ʒi

Tepid milk..............................℥ij

Inject one or two ounces three or four times a day. Used the above for many years with the most happy results. Wrote an article on that subject some years ago, and published in the *Texas Courier-Record*, January number, 1886.

Told her to call again and inform me how she got along.

On the 5th of December received a message to come and see her at the house. Found her in bed, lying with her knees raised; countenance expressing great pain; complains of much suffering in her back, in the lumbar region; weight in the hypogastrium; hands clutching the abdomen; violent headache, and vomiting. Her sister informs me that the medicine was given and directions were faithfully carried out, but no relief obtained. The girl is hysterical, and while there had one of her slight fits.

I soon realized that I had made a wrong diagnosis when she first called. I now made up my mind that she was suffering from obstruction of the cervical canal, and should have diognosed it when first called. "Open confession is good for the soul." More careful inquiry would, no doubt, have explained the situation. The patient not being in condition for examination, gave her an injection of $\frac{1}{4}$ grain of morphia sulph. On the 6th I saw her and was informed that she was somewhat relieved. Gave her another injection of morphia sulph., $\frac{1}{4}$ gr. On the 7th was informed that menstruation was ushered in, while in the act of defecation having taken some castor oil; the discharge, as usual, was scant, black, and very offensive, the result, no doubt, of the pent-up menses, but afforded much relief. Advised to continue the medicine and follow instructions, and come to the office when she got over her trouble, as soon after as convenient. On the 28th of January, 1891, after her usual painful experience, came to the office; made my first examination. Careful exploration reveals no appreciable cause for the trouble. No displacement; the uterus, adnexa, tubes and ovaries, normal; cervix hyperæmic and elongated. After much difficulty, a pin-hole os is discovered. I was now satisfied that I had before me a stenosis of the cervix, or some other pathological obstruction. The first indications were to open the lumen, which was attempted with a filiform bougie; but if it entered at all, was hardly appreciable. A small metal probe enters a little further. Not having things ready, tell her to come the next day, with the assurances that, in my opinion, she had a fair chance to get over her trouble if she had the patience. This was really said to give her some encouragement. On the 30th she returns.

In view of the overloaded medical literature on the subject of electricity, I confine myself to the technique of electrolysis in cases of stenosis of the cervix only, with the hope that it may prove acceptable to some unacquainted with the operation.

I use Le Clanche's battery, Dr. Robert Newman's electrodes. Mr. Tieman, of New York, made those of a smaller calibre. They are made on the metric scale, from one line up to No. 1, when Newman's are used.

The patient is in the semi-prone position; vagina sterilized in the usual manner, 1 to 3000 hyd. chloride.

All being ready, the electrode is connected with the negative pole, while the positive is attached to a flat sponge, which is pressed upon the abdomen, over the fundus uteri. A small electrode is introduced about six lines, which enters the os at about $\frac{1}{16}$ of an inch. I now remove the electrode and dip it in a campho-glycerine, bring it against the obstruction, and now complete the circuit, when the meter indicates five milliamperes. I gently hold it against the obstruction; in five minutes it slips into about $\frac{1}{12}$ of an inch, where arrested. Keep it there for six minutes, and it enters to $\frac{1}{2}$ an inch; is again arrested by some obstruction; hold it there, after three minutes that obstruction is overcome. I now take up a larger metal bulb and go over the same route, with little or no trouble. Be it remembered that the utmost gentleness is to be observed in the introduction of the electrodes. It is not pressure or pushing that gets the instrument to advance and through the obstruction. It is the chemical decomposition that disintegrates the adventitious tissues. Nothing is gained by driving; it is the galvanic current that does the business. I finish the seance, and tell my patient to return in five days.

On the 6th of February I commence with the last electrode, which enters with some difficulty, but after one minute contact slips in. I now take No. 1, which is just able to stretch the os. In five minutes it passes, but is arrested; hold the electrode against the obstruction, and augment the amperage to ten milliamperes. It gradually yields; complete the seance in sixteen minutes; to return in five days.

On the 12th of February she returns; introduce No. 1. electrode, which enters up to what I judge the internal os; increase the current up to ten milliamperes; in a few minutes it passes some obstruction. At the same seance a No. 5 passes with facility. On the 18th, repeat the operation with a No. 6 electrode, which enters readily. This seance is concluded in three minutes; to return in five days. Do not see her till the 28th of March. Reports as follows: Took the medicine prescribed; had for two days some pain, but much less than she usually suffered; was agreeably surprised to discover that during the night it must have begun to flow; in the morning found a brown discharge on her linen which, during the day, became pale red, lasting five days; had no fits, and tells me that she had a comparatively easy time of it.

The operation is repeated. A No. 6 electrode enters to about 2½ inches readily, and for the first time reaches the fundus uteri. To make a long story short, she remains well. She is under treatment up to April; has had fifteen applications, with the result that Miss M. is up to date, 1893, free from her past painful experience. More than two years have elapsed; has gone through her monthly periods without pain or inconvenience. She is enjoying good health; has gained flesh, and has only occasionally headache; not a trace of her former difficulty remains.

When it is considered that all this can be accomplished, without pain or inconvenience, is it not about time to dispense with the cutting, tearing and burning methods of the teachers?

The curing of the stenosis of the cervix uteri by galvanism is no longer a theory, it is an accomplished fact, being the most rational method, by far less dangerous, and certainly most gratifying in its general results.

By some accidental cause the therapeutic value of galvanism in these cases has been allowed to slumber. It is to emphasize and add my testimony to its advantages that this paper has been written.

In conclusion, one or two suggestions may not be out of place.

I follow in the manipulation and teaching of Dr. Robert Newman: Use as mild currents as is consistent with proper electrolytic action, avoiding carefully the caustic effects of strong currents.

It is important to remember, not to use the positive pole for the tip electrode, for narrowing of any kind. Should the mistake occur, the polarity of the electrode must be changed to the negative, and ascertain the fact before the electrode is removed, for the reason that there is a tendency for the metallic conductor to adhere. By changing the polarity, the tearing of the tissue is prevented.

For deviations, or flexion, the electrode must be bent to satisfy the direction of the canal.

